# Multimodal characterization of HLA-G expression across solid tumors reveals transcript-protein discordance and implications for immunotherapy targeting

**DOI:** 10.3389/fimmu.2026.1876556

**Published:** 2026-09-24

**Authors:** Alexandra Epp, Martha L. Serrano-Serrano, Florian Heil, Meher Majety, Abraham Silva, Julie Cheung, Mert Colpan, Nitya Nair

**Affiliations:** 1 Roche Pharma Research and Early Development, Early Development Oncology, Roche Innovation Center Munich, Penzberg, Germany; 2 Roche Pharma Research and Early Development, Early Development Oncology, Roche Innovation Center Basel, F. Hoffmann-La Roche Ltd., Basel, Switzerland; 3 Roche Pharma Research and Early Development, Discovery Oncology, Roche Innovation Center Munich, Penzberg, Germany; 4 Pharma Services Assay Development, Roche Tissue Diagnostics, Oro Valley, AZ, United States

**Keywords:** biomarker, companion diagnostic, HLA-G, immune checkpoint, immunotherapy, solid tumors, transcript-protein discordance, tumor microenvironment

## Abstract

**Introduction:**

Human leukocyte antigen-G (HLA-G) is a non-classical major histocompatibility complex class I molecule that functions as an immune checkpoint via inhibitory receptors such as immunoglobulin-like transcript 2 (ILT2), immunoglobulin-like transcript 4 (ILT4) and killer cell immunoglobulin-like receptor 2DL4 (KIR2DL4), suppressing anti-tumor immunity. Its prevalence and regulation across solid tumors remain incompletely defined due to low expression, spatial heterogeneity, and detection challenges.

**Methods:**

We performed a multimodal target prevalence analysis integrating data from a validated immunohistochemistry (IHC) assay for protein detection and prospective screening data from a phase 1 study of an HLA-G × CD3 T cell bispecific antibody (Clintrials.gov identifier: NCT05769959) with bulk RNA sequencing from early-phase oncology clinical trials.

**Results:**

Analysis of 252 patient tumor samples using an investigational HLA-G IHC assay showed that 59.9% were HLA-G positive, with highest prevalence in ovarian cancer (89%), non-small cell lung cancer (NSCLC) (76%), renal cell carcinoma (RCC) (71%), and pancreatic cancer (70%). Expression was generally low to moderate and spatially heterogeneous. Notably, discordance between transcript and protein expression was observed in melanoma, where high mRNA levels were not matched by detectable protein, suggesting post-transcriptional regulation. Prospective screening in a phase 1 cohort confirmed a high target positivity rate (77.5%). At the transcript level, HLA-G expression was enriched in inflamed tumors and correlated with interferon gamma (IFN-γ) signaling, antigen presentation pathways, and effector T-cell markers including cluster of differentiation 8 alpha (*CD8A*), interferon gamma gene (*IFNG*) and granzyme B (*GZMB*).

**Conclusions:**

HLA-G appears to be an inducible immune checkpoint associated with IFN-γ-driven immune activation and adaptive resistance. Transcript-protein discordance and spatial heterogeneity highlight the need for protein-level assessment in biomarker development. These findings inform companion diagnostic strategies for HLA-G-directed immunotherapies.

## 1 Introduction

Human leukocyte antigen-G (HLA-G) is a non-classical major histocompatibility complex (MHC) class I molecule with specialized immunoregulatory functions that promote immune tolerance. Under physiological conditions, HLA-G plays a central role at the maternal-fetal interface, where it suppresses cytotoxic activity of natural killer (NK) cells and T lymphocytes through engagement of inhibitory receptors such as immunoglobulin-like transcript 2 (ILT2), immunoglobulin-like transcript 4 (ILT4) and killer cell immunoglobulin-like receptor 2DL4 (KIR2DL4) expressed on immune and myeloid cells (1–3). Furthermore, the biological activity of HLA-G is tied to its spatial and cellular localization; it is the cell-surface, membrane-expressed molecule that directly interfaces with immune receptors to drive tolerance, while intracellular fractions (including endosomal pools) may not reflect functional ligand availability. In cancer, this tolerogenic pathway is frequently co-opted by tumor cells to evade immune surveillance, suppress effector T-cell function, and modulate antigen-presenting cells within the tumor microenvironment (2). Consistent with these immunosuppressive effects, HLA-G expression has been associated with adverse clinical outcomes across multiple tumor types (4–6). These features position HLA-G as an emerging immune checkpoint and a compelling target for immunotherapeutic strategies, including T cell-redirecting approaches such as T cell bispecific antibodies (TCBs) in solid tumors.

Despite increasing therapeutic interest, the prevalence and regulation of HLA-G across solid tumors remain poorly defined, largely due to challenges in its accurate detection. The high sequence homology (~86%) between HLA-G and classical HLA class I molecules (HLA-A, -B, and -C) confounds antibody-based detection and increases the risk of cross-reactivity (1, 7, 8). This is further compounded by the lack of standardized detection approaches and variability in antibody specificity, contributing to inconsistent assessment of HLA-G expression across studies (7, 9).

In addition, HLA-G is typically expressed at low levels and exhibits marked spatial heterogeneity within tumors, resulting in low signal-to-noise target detection ratios and substantial sampling bias. These technical challenges are compounded by biological complexity, including alternative splicing of the HLA-G transcript into at least seven isoforms (G1-G4 membrane-bound and G5-G7 soluble), which may be differentially detected depending on assay specificity (7). Together, these factors have contributed to substantial variability and inconsistency in reported HLA-G prevalence across tumor types and studies, with reported prevalence varying widely even within the same indication depending on methodology and cohort selection (9, 10).

Methodological differences in tissue sampling further exacerbate these challenges. Analyses based on tissue microarrays (TMAs) have yielded inconsistent estimates of HLA-G prevalence, likely reflecting the inability of small core biopsies to capture focal and heterogeneous expression patterns, underscoring the importance of whole-tissue section analysis. These issues are exemplified in recent work describing the HLA-G-directed T cell engager JNJ-78306358, in which target prevalence was primarily assessed using TMA-based approaches (11). In that study, the authors acknowledge key limitations, including the inability to detect HLA-G in formalin-fixed paraffin-embedded (FFPE) tissue and the restricted sampling inherent to TMA analysis, both of which may underestimate expression in heterogeneous tumors (11).

Recent clinical evaluation of HLA-G-targeted therapies, including first-in-human studies of HLA-G × CD3 T cell bispecific antibodies, has provided initial insights into the safety profile, pharmacodynamic activity, and therapeutic potential of targeting HLA-G in solid tumors (12). In these studies, HLA-G expression was detected in a subset of archival tumor samples, and treatment was associated with peripheral T-cell activation. While these findings support the biological relevance of HLA-G as a target, they also highlight the need for a more comprehensive understanding of its prevalence, regulation, and spatial distribution within tumors. In this context, the present study builds on these observations by integrating transcriptomic and protein-level analyses with prospective clinical screening to more systematically define HLA-G expression and its immune context across solid tumors.

At a mechanistic level, emerging evidence suggests that HLA-G expression is not constitutive but dynamically regulated within the tumor microenvironment. Associations with interferon gamma (IFN-γ)-related pathways, antigen presentation machinery, and effector T-cell signatures support a model in which HLA-G is induced as part of an adaptive immune resistance program, analogous to established immune checkpoints such asprogrammed death-ligand 1 (PD-L1) (3, 12). In this context, HLA-G expression may reflect ongoing immune pressure while simultaneously contributing to feedback inhibition of anti-tumor immunity. Notably, prior studies have demonstrated that HLA-G mRNA expression does not consistently translate to detectable protein levels, highlighting the importance of post-transcriptional and post-translational regulation and raising concerns regarding the use of transcriptomic data alone to define target prevalence (9, 12, 13).

Here, we sought to define the landscape, regulation, and clinical relevance of HLA-G expression across solid tumors using a multimodal approach. By integrating bulk RNA sequencing with a validated, FFPE-compatible immunohistochemistry (IHC) assay, we characterize HLA-G expression at both transcript and protein levels and define its association with immune context within the tumor microenvironment. We further extend these findings through prospective evaluation of HLA-G expression in patients enrolled in a phase 1 clinical trial of an HLA-G × CD3 T cell bispecific antibody (ClinTrials.gov identifier: NCT05769959), enabling assessment of target prevalence in a clinically relevant setting.

We hypothesize that HLA-G represents an inducible immune checkpoint linked to interferon-driven immune activation, and that protein-level assessment is required to accurately capture its functional expression in tumors. By reconciling transcript-protein relationships and addressing key methodological limitations of prior studies, this work establishes a mechanistic and translational framework for HLA-G as a biomarker and therapeutic target and provides a foundation for patient selection strategies in HLA-G-directed immunotherapy.

## 2 Materials and methods

### 2.1 Patient cohorts

To evaluate HLA-G protein expression, FFPE tumor tissue sections were obtained from Roche Tissue Diagnostics (Tucson, AZ, USA), as well as from 40 patients screened for eligibility in an open-label, multicenter phase 1 clinical trial evaluating an HLA-G × CD3 TCB (ClinicalTrials.gov identifier: NCT05769959). Patient tumor samples from this clinical trial cohort were collected during the initial screening window, prior to any therapeutic administration of the HLA-G × CD3 TCB.

Normal tissue expression and assay specificity were further assessed using a commercially sourced multi-tissue microarray comprising normal (Tour of Body, TOB) and tumor (Tour of Tumor, TOT) cores (Novus Biologicals; Centennial, CO, USA, catalog number NBP2-30233, lot BC10), encompassing a broad range of normal and malignant tissues.

To evaluate HLA-G transcript expression across human malignancies, bulk RNA sequencing (RNA-seq) data were obtained from a harmonized data mart integrating ten phase 1-phase 2 cancer immunotherapy (CIT) trials sponsored by F. Hoffmann-La Roche. The dataset comprised approximately 600 patients across eleven solid tumor indications including CD8 immune phenotype classification [IHC combined density score as previously described (14)] and prior checkpoint inhibitor (CPI) treatment status as naive or experienced, the latter if any previous therapy includes approved CPI (median post-CPI: 65 days). Among CPI-experienced patients, prior immunotherapy consisted of nivolumab (54.1%), pembrolizumab (22.5%), atezolizumab (15.0%), or other approved CPI (8.4%).

### 2.2 Immunohistochemistry assay development and validation

An investigational IHC assay was developed using a previously characterized, commercially available mouse monoclonal antibody (clone 4H84, Abcam) targeting the HLA-G alpha domain common to all membrane-bound HLA-G isoforms (G1–G4). The assay was optimized and analytically validated for use on FFPE tissues using the BenchMark ULTRA platform (Ventana Medical Systems, Tucson, AZ, USA) in combination with the OptiView DAB detection system. Final staining conditions included a working antibody concentration of 12.5 micrograms per milliliter, epitope retrieval using CC1 buffer for 80 minutes, and antibody incubation for 32 minutes. Analytical validation included assessment of precision, specificity, sensitivity, and robustness. Specificity was evaluated using cell lines with defined HLA expression profiles, as well as TOB and TOT arrays to assess on-target staining, off-target binding, background signal, and staining artifacts. Reproducibility was assessed through inter-day, inter-instrument, and inter-operator studies, in which four tumor samples were stained across four independent instruments, on four non-consecutive days, by four different operators. Concordance was evaluated by a pathologist, with at least 90 percent agreement required for assay acceptance.

### 2.3 Cell lines and specificity controls

To assess IHC assay specificity for HLA-G, JEG3 cells were used as a positive control due to their reported constitutive expression of HLA-G. As a negative control, the cell line K562 was employed due to their known lack of endogenous HLA-G expression. HEK293T cell lines were engineered to overexpress individual HLA class I molecules, including HLA-A, HLA-B, HLA-C, HLA-E, HLA-F, and HLA-G. In addition, U266, HEL and HEK293 cell lines were included due to their known endogenous expression of HLA-F based on mRNA expression data (Broad Institute Cancer Cell Line Encyclopedia (CCLE) database, https://sites.broadinstitute.org/ccle/). These cell line panels were used to evaluate antibody specificity and potential cross-reactivity during assay development (**Supplementary Table 1**).

### 2.4 Immunohistochemistry staining and scoring

Patient-derived FFPE tumor sections were stained using the investigational HLA-G IHC assay at the College of American Pathologists (CAP)/Clinical Laboratory Improvement Amendments (CLIA)-accredited laboratory of Roche Tissue Diagnostics (Tucson, AZ, USA). Staining was evaluated by a board-certified pathologist for both cytoplasmic and membranous signals. HLA-G expression was quantified using an intensity-weighted H-score (range 0–300), calculated as: *H-score = (percent tumor cells at 1+) × 1 + (percent tumor cells at 2+) × 2 + (percent tumor cells at 3+) × 3.* Staining intensity was categorized as weak (1+), moderate (2+), or strong (3+). Both membranous and cytoplasmic staining patterns were considered in scoring.

Tumors were classified as HLA-G positive if at least 1 percent of tumor cells demonstrated weak (1+) or greater staining intensity. H-score distributions were further stratified into low (1–100), intermediate (101–200), and high (201–300) expression categories for downstream analyses.

### 2.5 Evaluation of normal tissue expression

To characterize baseline HLA-G expression in non-malignant tissues, normal tissue arrays were evaluated using the investigational IHC assay across a broad panel of organs.

### 2.6 Tumor bulk RNA sequencing and data processing

Genomic DNA and total RNA were simultaneously purified from FFPE tissue sections using a DNA/RNA FFPE extraction kit; during RNA processing, the RNA-containing supernatant was heated to partially reverse formalin-induced crosslinks prior to extraction, and resulting RNA was assessed for quantity and quality before library preparation. Bulk RNA sequencing was performed on FFPE tumor specimens using the TruSeq RNA Exome Kit (Illumina). Library preparation included polymerase chain reaction (PCR) amplification (15 cycles pre- and post-hybridization capture). Libraries were quality-controlled using quantitative PCR and TapeStation analysis prior to sequencing on a NovaSeq 6000 platform (Xp workflow, 50 bp paired-end reads, with a minimum depth of 40 million reads per sample). RNA-sequencing data were analyzed using HTSeqGenie (15) within the Bioconductor framework (16). Reads with low nucleotide quality (≥70% of bases with quality <23) or matching rRNA and adapter sequences were removed. The remaining reads were aligned to the human reference genome (GRCh38.p10) using GSNAP (version ‘2013-10-10-v2’), allowing a maximum of two mismatches per 75-base sequence (17). Transcript annotation was based on the GENCODE database (release 27). Gene expression levels were quantified by counting reads mapping unambiguously to exonic regions of each gene. Gene signatures were scored using the BioQC R package (18), deploying a previously validated interferon gamma gene (*IFNG*) gene expression signature alongside a curated panel of relevant immune-related genes selected based on established literature [(18, 19); **Supplementary Table 4**)].

### 2.7 Statistical analysis

Descriptive statistics were used to summarize HLA-G expression at both transcript and protein levels across tumor types. Associations between RNA and protein expression, as well as relationships with clinical and immune parameters, were explored using standard statistical methods as described in the relevant sections. Differences in transcript expression between clinical cohorts were evaluated using the two-sample Wilcoxon test.

## 3 Results

### 3.1 Analytical validation demonstrated specificity of the investigational HLA-G IHC assay

An investigational IHC assay targeting HLA-G was developed using a previously characterized, commercially available mouse monoclonal antibody (clone 4H84, Abcam) directed against the alpha 1 domain, which is present across all membrane-bound HLA-G isoforms (G1–G4). Although clone 4H84 has been used in prior HLA-G studies, its performance had not previously been analytically validated for quantitative, clinically deployable use on FFPE tissue; the present work therefore focused on optimizing and validating a standardized IHC assay incorporating this antibody, rather than on *de novo* characterization of the antibody itself. Specificity testing in engineered cell line systems demonstrated staining restricted to HLA-G-expressing cells (**Supplementary Figure 1**), with no detectable cross-reactivity to classical HLA class I molecules (HLA-A, HLA-B, HLA-C) or HLA-E (**Supplementary Table 1**). Weak staining (8%) observed in artificial HLA-F-overexpressing HEK293T cells (**Supplementary Figure 1**) was not reproduced in cell lines with endogenous HLA-F expression (U266, HEL and HEK293), supporting assay specificity under physiologically relevant target densities (**Supplementary Table 1**).

Evaluation across a broad panel of normal tissues demonstrated minimal staining across organs, with expected expression largely confined to placental tissue (**Supplementary Table 2**). In non-placental organs, low-level physiological expression was highly restricted; for instance, the sublingual salivary gland exhibited weak, focal, linear membranous HLA-G reactivity within acinar and ductal epithelial structures, while the spleen demonstrated patchy, non-contiguous immunoreactivity confined to scattered cell subsets within the perifollicular/marginal zones (**Supplementary Table 2**). Rare immune cell staining was observed at low levels, consistent with known biology. These data establish a robust platform for detecting low-abundance and spatially heterogeneous HLA-G protein in FFPE tumor specimens.

### 3.2 HLA-G protein expression is common but low and spatially heterogeneous

Application of the validated assay across 252 tumor samples demonstrated that HLA-G protein expression is a frequent feature of solid tumors (**Figure 1**). Overall, 59.9% of tumors were classified as HLA-G positive (**Table 1**). Prevalence varied across indications, with enrichment in ovarian cancer (89%), non-small cell lung cancer (NSCLC) (76%), renal cell carcinoma (RCC) (71%), and pancreatic cancer (70%), whereas colorectal cancer (CRC) (36%) and melanoma (17%) showed lower positivity rates (**Table 1**; **Figure 1**).

**Figure 1:**
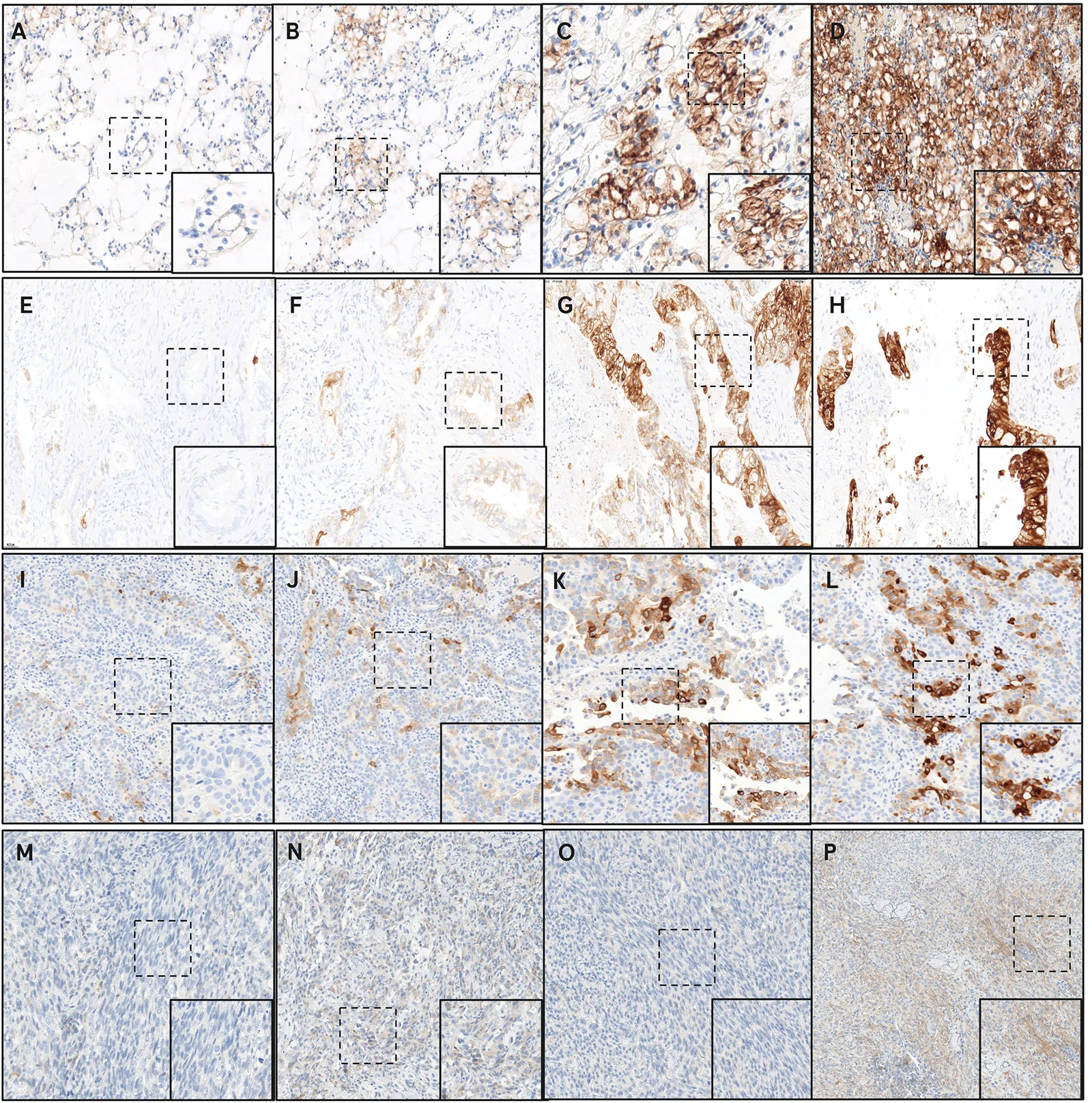
Representative immunohistochemical images from HLA-G immunohistochemistry (IHC) assay validation and prevalence analyses demonstrating HLA-G protein expression in tumors from renal cell carcinoma (RCC), pancreatic cancer, ovarian carcinoma and melanoma patients. **(A–D)** Tumor samples from RCC patients across H-score categories. **(A)** H-score 0 indicates no detectable HLA-G expression in tumor cells. **(B)** H-score 1+ corresponds to low or weak expression. **(C)** H-score 2+ represents moderate expression. **(D)** H-score 3+ denotes strong expression within clear tumor cells. Staining is predominantly cytomembranous with additional cytoplasmic signals in higher intensity cases. **(E–H)** Tumor samples from pancreatic cancer patients across H-score categories. **(E)** H-score 0 indicates no detectable expression. **(F)** H-score 1+ corresponds to weak expression. **(G)** H-score 2+ represents moderate expression within glandular tumor structures. **(H)** Strong expression (H-score 3+) is observed in additional pancreatic cancer tumors, characterized by prominent cytomembranous staining of glandular epithelial cells. **(I–L)** Tumor samples from ovarian cancer patients across H-score categories. **(I)** An H-score of 0 demonstrates a total absence of detectable HLA-G in the tumor cells. **(J)** H-score 1+ represents a weak or faint staining pattern, **(K)** H-score 2+ signifies a moderate degree of expression, and **(L)** H-score 3+ indicates highly intense HLA-G positivity within the papillary structures and malignant nests, showing clear localization along both the cell membranes and cytoplasm of the neoplastic epithelium. **(M–P)** Tumor samples from two melanoma patients displaying negative (0) and weak (1+) H-scores. In the first case, **(M)** demonstrates a total absence of detectable HLA-G expression (H-score 0) and **(N)** represents weak (1+) expression within the malignant melanocytes. In the second case, **(O)** similarly shows no detectable immunoreactivity (H-score 0) and **(P)** denotes weak (1+) positivity, with the staining localized along the cell membranes and cytoplasm of the neoplastic cells within the tumor nests. Images were derived from HLA-G IHC prevalence and validation datasets. Panoramic overview and high-magnification inset images were captured at 20× and 40× magnification, respectively.

**Table 1:** HLA-G expression by IHC across patient tumors.

Indication	No. patients	Positive cases n (%)	H-score		
			1–100 n (%)	101–200 n (%)	201–300 n (%)
Ovarian cancer	45	40 (89%)	40 (89%)	0 (0%)	0 (0%)
NSCLC	41	31 (76%)	29 (71%)	2 (5%)	0 (0%)
RCC	42	30 (71%)	23 (55%)	6 (14%)	1 (2%)
Pancreatic cancer	40	28 (70%)	21 (53%)	7 (18%)	0 (0%)
CRC	42	15 (36%)	15 (36%)	0 (0%)	0 (0%)
Melanoma	42	7 (17%)	7 (17%)	0 (0%)	0 (0%)
**All indications**	**252**	**151 (59.9%)**	**135 (53.6%)**	**15 (6.0%)**	**1 (0.4%)**

CRC, colorectal cancer; IHC, immunohistochemistry; NSCLC, non-small cell lung cancer; RCC, renal cell carcinoma. Values in the "All indications" row represent the total number of patients and combined percentages across all listed cancer indications.

Despite this broad prevalence, expression levels were generally low, with the majority of positive tumors exhibiting H-scores within the 1–100 range (**Table 1**; **Figure 1**). Only a small fraction of tumors demonstrated higher H-scores above 100 (**Table 1**). Representative IHC images demonstrate tumor cyto-membrane staining with variable intensity and spatial distribution (**Figure 1**), indicating that HLA-G expression is heterogeneous within and across tumor types.

### 3.3 Tumor-type-specific expression patterns highlight spatial and biological heterogeneity

Further inspection of tumor-specific patterns revealed substantial heterogeneity in both prevalence and expression levels (**Table 1**). Pancreatic cancer and RCC exhibited the broadest dynamic range of expression, including cases with intermediate-to-high cyto-membrane staining (H-scores 101–200 in 18% of pancreatic cancer and 14% of RCC, with one RCC case scoring 201-300; **Table 1**). In contrast, ovarian carcinoma showed a narrower expression profile: although nearly all cases were positive (89%), staining was uniformly low (H-scores 1–100 only, with no 101–200 or 201-300; **Table 1**). CRC and melanoma demonstrated minimal expression overall (**Table 1**), suggesting tumor-type-specific regulation of HLA-G. In addition, marked intratumoral heterogeneity was observed within positive cases, with focal regions of positive staining interspersed with negative areas (**Figure 1**), highlighting the importance of whole-tissue assessment.

### 3.4 Prospective clinical screening confirms high target prevalence

To assess clinical applicability, the validated assay was applied prospectively in a phase 1 clinical trial cohort (**Table 2**). Overall, 77.5% of patients were classified as HLA-G positive (**Table 2**), consistent with retrospective analyses. Most tumors exhibited low-to-moderate expression (**Figure 2A**), while a subset demonstrated higher H-scores, particularly in ovarian carcinoma and pancreatic cancer (**Figures 2A–H**; **Table 2**). These findings highlight inter and intra patient heterogeneity in HLA-G expression.

**Table 2:** HLA-G expression prospectively evaluated by IHC in a phase 1 patient screening cohort.

Indication	No. patients	Positive cases n (%)	H-score		
			1–100 n (%)	101–200 n (%)	201–300 n (%)
CRC	2	2 (100%)	2 (100%)	0 (0%)	0 (0%)
NSCLC	7	6 (86%)	5 (71.4%)	0 (0%)	1 (14.3%)
Ovarian cancer	8	8 (100%)	6 (75%)	0 (0%)	2 (25%)
Pancreatic cancer	20	13 (65%)	6 (30%)	4 (20%)	3 (15%)
RCC	3	2 (66.7%)	2 (66.7%)	0 (0%)	0 (0%)
**All indications**	**40**	**31 (77.5%)**	**21 (52.5%)**	**4 (10%)**	**6 (15%)**

CRC, colorectal cancer; NSCLC, non-small cell lung cancer; RCC, renal cell carcinoma; IHC, immunohistochemistry. Values in the "All indications" row represent the total number of patients and combined percentages across all listed cancer indications.

**Figure 2:**
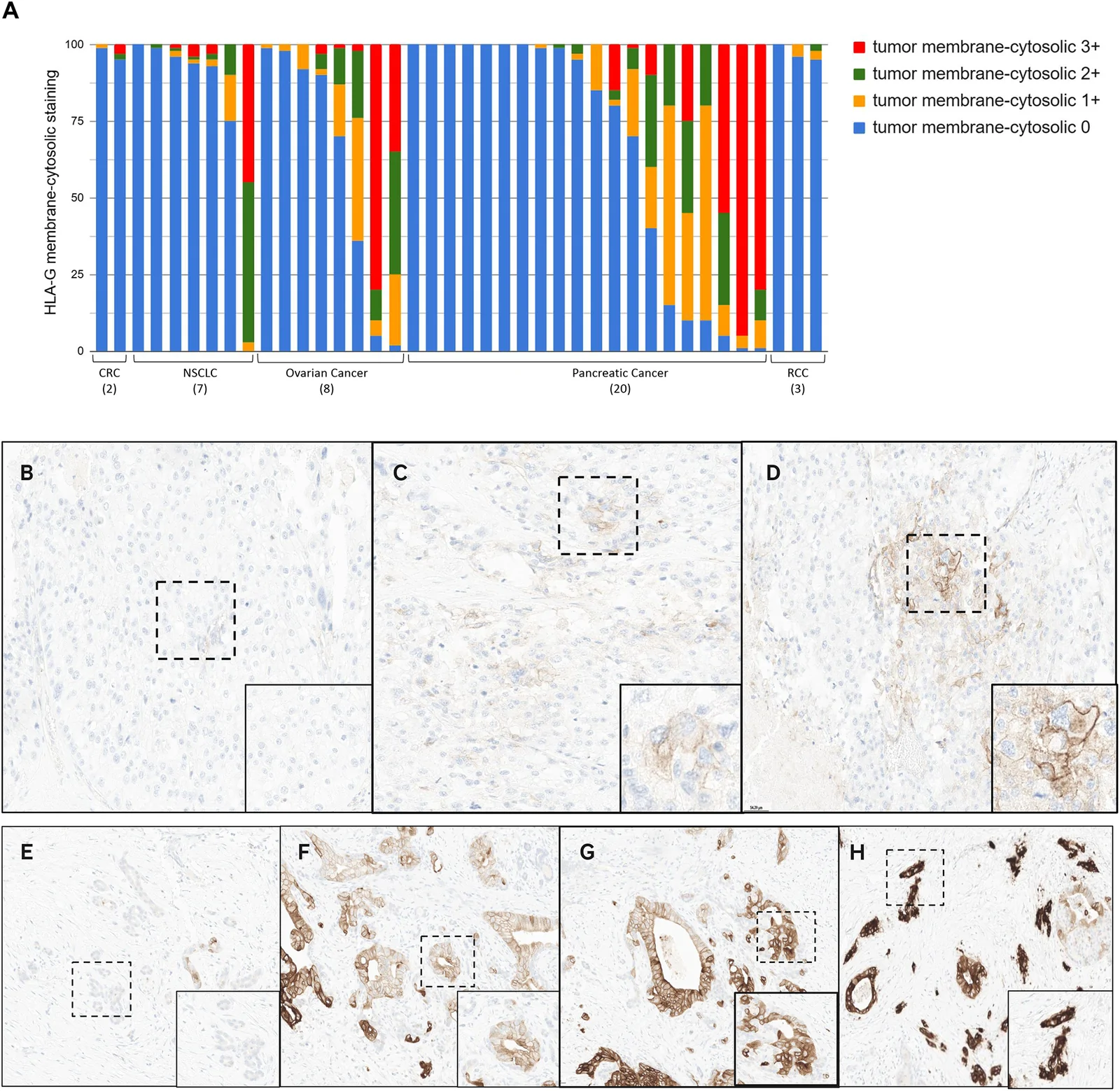
HLA-G protein expression in the phase 1 clinical study cohort. **(A)** Distribution of HLA-G tumor cell cyto-membrane staining across individual patients in the phase 1 study population, grouped by tumor indication. Stacked bar plots show the proportion of tumor cells at staining intensities 0 (no expression), 1+ (weak), 2+ (moderate), and 3+ (strong) for each sample, illustrating inter-patient variability and predominance of low to moderate expression across indications. **(B–D)** Representative immunohistochemical staining of HLA-G in tumors from RCC patients screened in the phase 1 study. **(B)** H-score 0 indicates no detectable expression. **(C)** H-score 1+ corresponds to weak expression. **(D)** H-score 2+ represents moderate expression within tumor cells. Strong expression (H-score 3+) was not observed in RCC tumors. Insets highlight representative regions of interest. **(E–H)** Representative immunohistochemical staining of HLA-G in tumors from pancreatic cancer patients screened in the phase 1 study. **(E)** H-score 0 indicates no detectable expression. **(F)** H-score 1+ corresponds to weak expression. **(G)** H-score 2+ represents moderate expression. **(H)** H-score 3+ denotes strong expression within glandular epithelial tumor structures, with prominent cytomembranous staining. Insets highlight representative regions of interest. Overview and inset images were captured at 20× and 40× magnification, respectively. CRC, colorectal cancer; NSCLC, non-small cell lung cancer; RCC, renal cell carcinoma.

### 3.5 HLA-G expression differs between primary and metastatic tumors

To evaluate the impact of disease stage, HLA-G expression was compared between primary and metastatic tumors obtained from both assay validation cohorts and prospective clinical screening from patients with available metadata (**Table 3**). Across indications, patterns varied, indicating context-dependent regulation. CRC and melanoma exhibited consistently low expression in both primary and metastatic samples (**Table 3**). In NSCLC and RCC, broader variability was observed in primary tumors, with lower median expression in metastatic lesions (**Table 3**). In contrast, ovarian carcinoma showed a marked enrichment of HLA-G expression in metastatic disease: median H-score increased from 4 in primary tumors (n=17; range 0–90) to 35 in metastatic lesions (n=36; range 0–265), with a substantially wider dynamic range and a higher proportion of cases reaching intermediate-to-strong staining levels (**Table 3**). Pancreatic cancer showed comparable expression between primary and metastatic settings (median H-score 15 vs 15.5; **Table 3**), indicating relative stability across disease progression. Of note, only 3–4 metastatic cases were available for CRC and RCC, limiting comparisons for these tumors.

**Table 3:** HLA-G expression by IHC in primary versus metastatic patient tumors from the assay validation cohorts and phase 1 study patients with available metadata.

Indication	Setting	No. patients	H-score range	Median H-score
CRC	Primary	40	0–31	0
CRC	Metastatic	4	0–22	0
Melanoma	Primary	20	0–5	0
Melanoma	Metastatic	22	0–20	0
NSCLC	Primary	24	0–242	4
NSCLC	Metastatic	24	0–115	1
Ovarian cancer	Primary	17	0–90	4
Ovarian cancer	Metastatic	36	0–265	35
Pancreatic cancer	Primary	43	0–269	15
Pancreatic cancer	Metastatic	14	0–289	15.5
RCC	Primary	42	0–205	13
RCC	Metastatic	3	0–10	7

Primary or metastatic annotation was available for 289 of the 292 tumors included in **Tables 1** and **2**; three pancreatic cancer samples were excluded from this analysis because primary or metastatic status was not available. CRC, colorectal cancer; NSCLC, non-small cell lung cancer; RCC, renal cell carcinoma. ; IHC, immunohistochemistry

### 3.6 HLA-G transcript expression reveals low abundance and high heterogeneity

Bulk RNA sequencing analysis demonstrated that HLA-G transcripts are broadly detectable across tumor types (**Figure 3**). However, absolute expression levels were low, with 7 of 11 indications showing a median log2 transcripts per million (TPM) value below 2 (**Supplementary Table 3**). Substantial variability was observed within each tumor type, with expression spanning wide dynamic ranges (**Figure 3**; **Supplementary Table 3**). RCC and melanoma exhibited relatively higher median expression, whereas breast and head and neck cancer showed lower median levels. These findings indicate that HLA-G expression at the transcript level is heterogeneous and not constitutively high.

**Figure 3:**
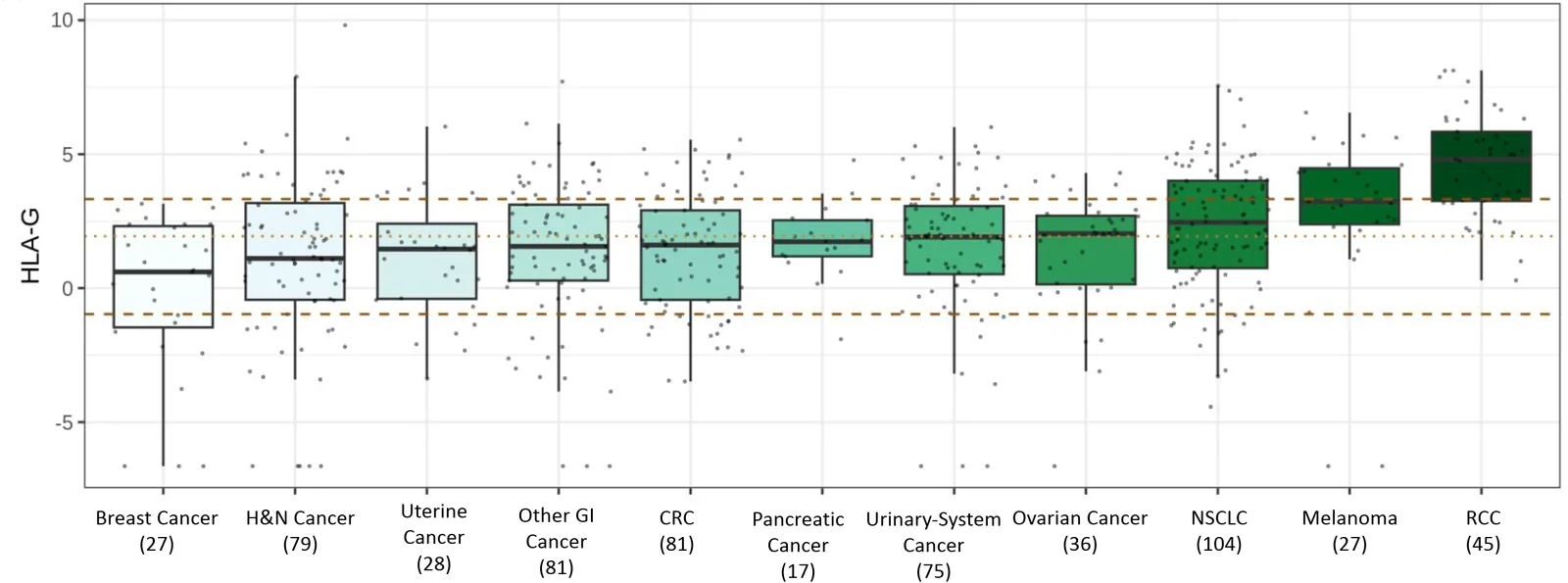
HLA-G transcript expression across tumor indications. HLA-G transcript expression measured by RNA sequencing in patient tumors according to indication. Boxplots show log2-transformed TPM values for individual samples within each indication, with overlaid points representing individual patient tumors. The central line indicates the median, and boxes represent the interquartile range. Dashed lines denote thresholds for no expression and low expression (log2(10 TPM) = 3.32). Sample numbers per indication are shown in parentheses. Across indications, HLA-G expression is generally low but highly variable, with relatively higher expression observed in RCC, melanoma and NSCLC compared with breast cancer, CRC, and other gastrointestinal (GI) cancers. CRC, colorectal cancer; H&N, head and neck; NSCLC, non-small cell lung cancer; RCC, renal cell carcinoma; TPM, transcripts per million.

### 3.7 HLA-G expression is associated with an inflamed tumor microenvironment

Gene-level analysis showed co-expression of *HLA-G* with immune-related genes including *HLA-A*, *HLA-B*, leukocyte immunoglobulin-like receptor B1 (*LILRB1*), and leukocyte immunoglobulin-like receptor B2 (*LILRB2*) (**Figure 4A**). Pathway-level analysis further demonstrated enrichment of antigen presentation, IFN-γ signaling, and effector T-cell signatures in tumors with higher HLA-G expression (**Figure 4A**). In RCC in particular, HLA-G transcript expression was higher in CD8-inflamed tumors compared with desert tumors (**Figure 4B**). Given substantial intra-group variability and limited sample size in several indications, these comparisons were not subjected to formal statistical testing except in indications with sufficient sample size per CD8 phenotype group (n>10; head and neck, other gastrointestinal and urinary system cancers and NSCLC); within this subset, the difference between inflamed and desert phenotypes reached statistical significance in head and neck cancer (two-sample Wilcoxon test, P<0.05), while the remaining comparisons were not statistically significant. These patterns should therefore be interpreted as descriptive, exploratory trends rather than statistically validated associations. In addition, in several tumor types, including other gastrointestinal malignancies, CRC, and ovarian cancer, the relationship between immune phenotype (desert versus inflamed) and HLA-G transcript expression was inconsistent or not apparent; notably, ovarian carcinoma did not show a clear association between immune phenotype and HLA-G mRNA expression despite comparatively elevated HLA-G protein levels relative to other tumor types (**Table 1**), a discrepancy that is addressed further in the Discussion. These findings indicate that HLA-G expression is linked to coordinated immune activation programs in a subset of tumor types, although this association is not uniform across all indications profiled and should not be generalized. Consistent with this, correlation analyses demonstrated positive associations between *HLA-G* and cluster of differentiation 8 alpha (*CD8A*), *IFNG*, *HLA-A* and granzyme B (*GZMB*) (**Figure 5**; indications with n>30) across most tumor types, although this relationship was not consistent across all malignancies: other gastrointestinal cancers showed a weak inverse association between HLA-G expression and both *CD8A* and *IFNG*. These relationships were strongest in RCC, NSCLC, and head and neck and weaker in CRC and other gastrointestinal cancers, indicating tumor-type-specific coupling between HLA-G expression and immune activation.

**Figure 4:**
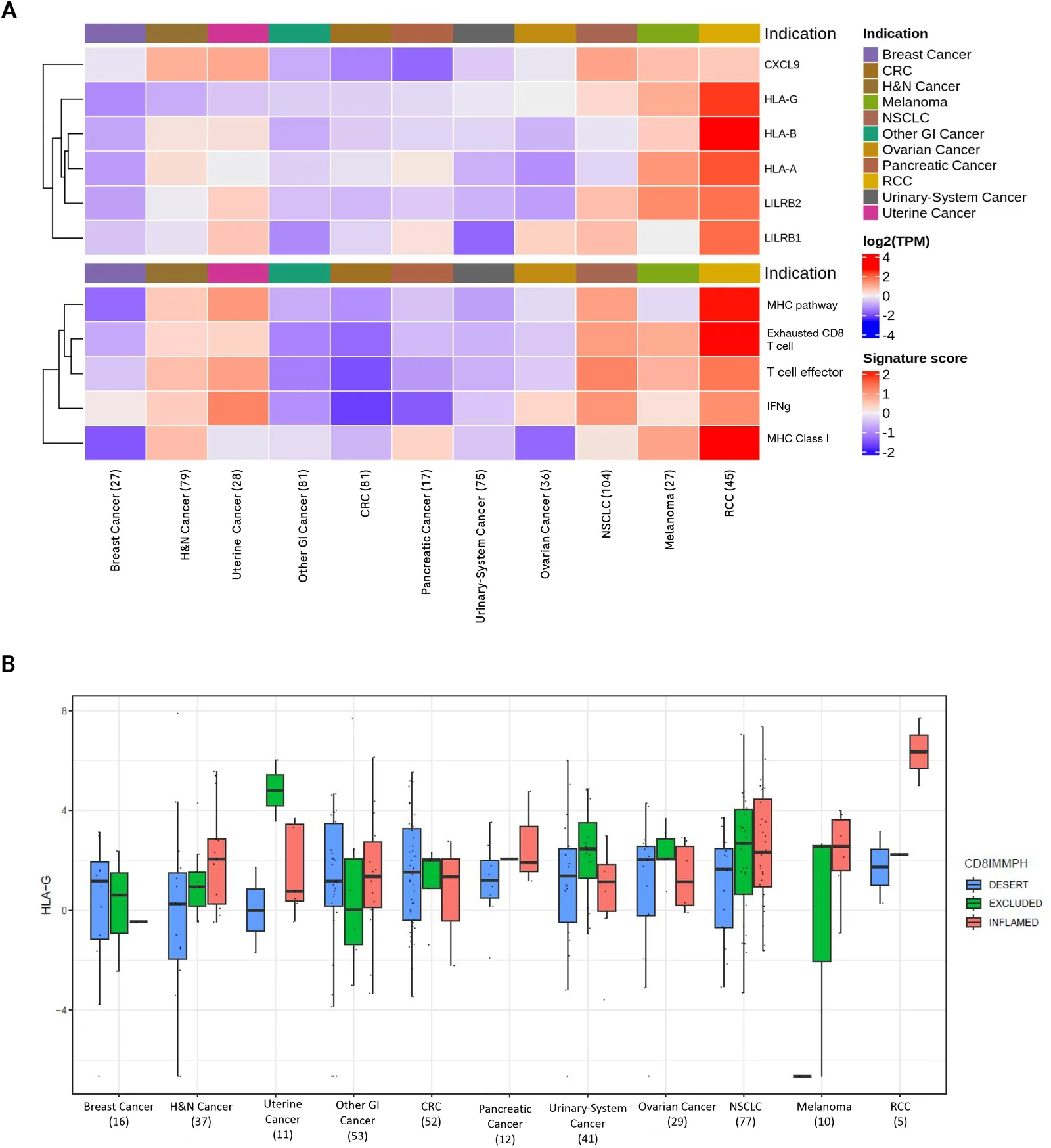
Association of HLA-G gene expression with immune-related genes, pathways, and tumor immune phenotypes. **(A)** Heatmap showing gene-level and pathway-level associations of *HLA-G* gene expression in patient tumors according to indication. Upper panel displays expression (log2 TPM) of *HLA-G* alongside antigen presentation genes (*HLA-A*, *HLA-B*) and its inhibitory receptors (*LILRB1*, *LILRB2*), as well as *CXCL9*. Lower panel shows enrichment of immune-related pathway signatures, including MHC class I antigen presentation (MHC pathway), exhausted CD8 T cell signature (Exhausted CD8 T cell), T effector gene signature (T cell effector), interferon gamma response (IFNg), and MHC class I pathway score (MHC Class I); signature gene lists are given in **Supplementary Table 4**. Sample numbers per indication are shown in parentheses. Tumor types are annotated by indication, highlighting coordinated upregulation of HLA-G with immune activation programs, particularly in RCC, melanoma, and NSCLC. **(B)** HLA-G transcript expression in patient tumors stratified by tumor immune phenotype (desert, excluded, inflamed) and indication. Boxplots show log2 TPM values with individual samples overlaid; sample numbers per indication are shown in parentheses. Across multiple tumor types, HLA-G expression is enriched in inflamed tumors relative to immune desert phenotypes, with intermediate levels observed in immune-excluded tumors. This pattern supports an association between HLA-G gene expression and immune activation within the tumor microenvironment. CD8IMMPH, CD8 immune phenotype; CRC, colorectal cancer; CXCL9, C-X-C motif chemokine ligand 9; GI, gastrointestinal; H&N, head and neck; HLA-A, human leukocyte antigen A; HLA-B, human leukocyte antigen B; LILRB1, leukocyte immunoglobulin-like receptor B1; LILRB2, leukocyte immunoglobulin-like receptor B2; MHC, major histocompatibility complex; NSCLC, non-small cell lung cancer; RCC, renal cell carcinoma; TPM, transcripts per million.

**Figure 5:**
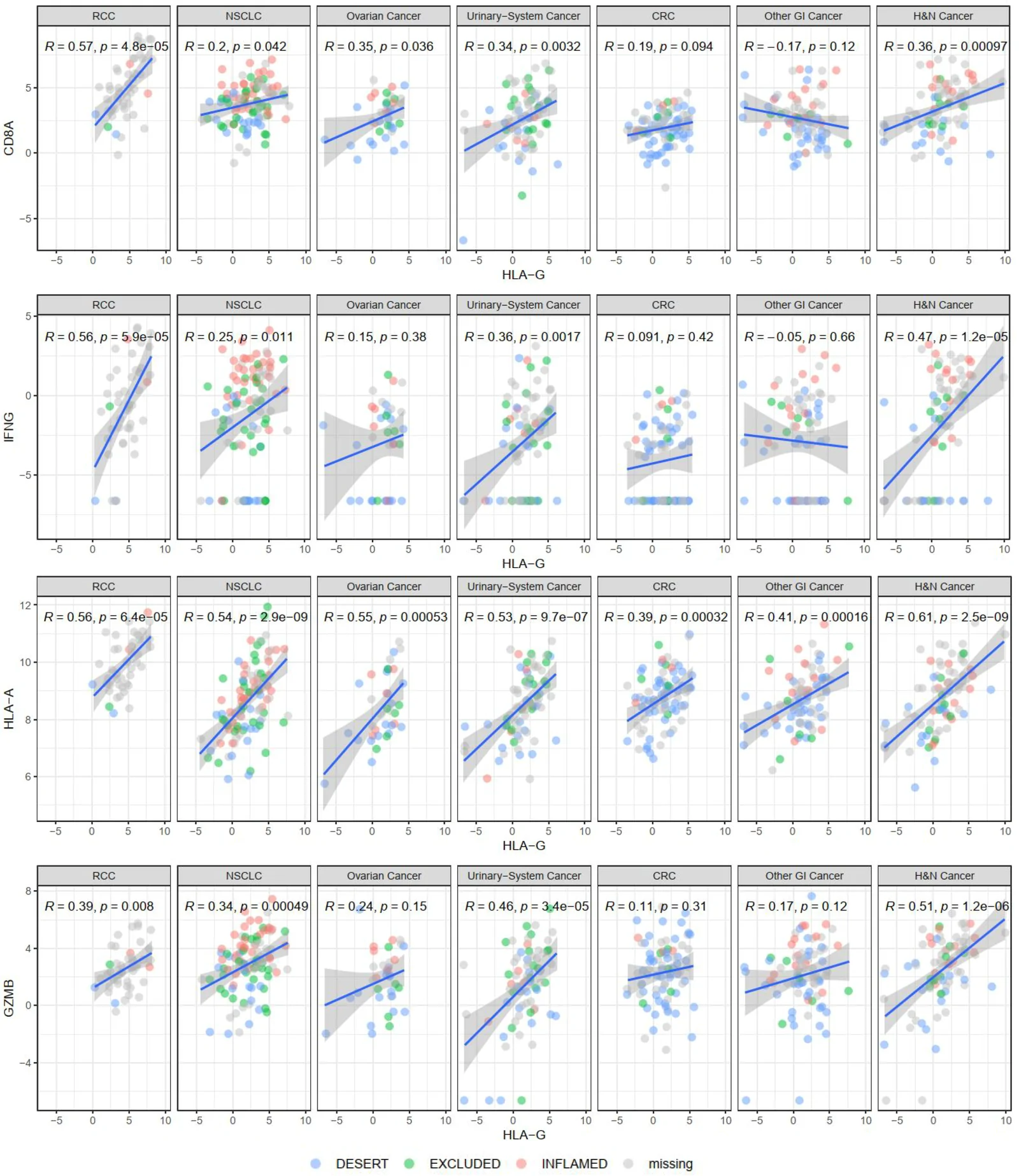
Tumor-type-specific associations between HLA-G expression and immune activation markers. Scatter plots showing relationships between HLA-G transcript expression and immune-related genes across tumor indications, including RCC, NSCLC, ovarian, urinary-system, CRC, other GI, and H&N cancers. Indications with fewer than 30 samples are not shown; sample numbers per indication are given in **Supplementary Table 3**. Each point represents an individual patient tumor sample and is colored according to CD8 immune phenotype (desert, excluded, inflamed, or missing). Blue lines indicate linear regression fits with shaded confidence intervals. Pearson correlation coefficients (R) and corresponding p-values are shown within each panel. The top row shows the association of *HLA-G* with *CD8A*, the second row the association with *IFNG*, the third row the association with *HLA-A*, and the bottom row the association with *GZMB*. Across tumor types, HLA-G demonstrates variable but generally positive relationships with immune activation markers, with the most consistent and strongest correlations observed with *HLA-A*. CD8A, cluster of differentiation 8 alpha; CRC, colorectal cancer; GI, gastrointestinal; GZMB, granzyme B; H&N, head and neck; HLA-A, human leukocyte antigen A; IFNG, interferon gamma gene; NSCLC, non-small cell lung cancer; RCC, renal cell carcinoma.

HLA-G expression did not differ in tumors from patients who were CPI-naive as compared to CPI-experienced patients across indications (**Supplementary Figure 2**), albeit the analysis was not conducted in paired biopsies from the same patients pre and post CPI-treatment.

### 3.8 Transcript-protein discordance indicates post-transcriptional regulation

Comparison of transcriptomic and protein data revealed discordance between HLA-G mRNA and protein expression, in melanoma, where relatively high transcript levels were observed despite low protein expression (**Table 1**; **Figure 3**; **Supplementary Table 3**).

In contrast, ovarian cancer, NSCLC, RCC, and pancreatic cancer showed concordance between transcript and protein expression. These findings suggest that HLA-G expression is subject to post-transcriptional and post-translational regulation, limiting protein expression in certain tumor contexts.

Collectively, these data support a model in which HLA-G functions as an inducible immune checkpoint associated with IFN-γ-driven immune activation, with expression regulated at multiple levels and varying across tumor types.

## 4 Discussion

This study provides a comprehensive and multimodal characterization of HLA-G expression across human solid tumors. Our findings confirm high target prevalence in ovarian cancer, NSCLC, RCC, and pancreatic cancer, and demonstrate strong concordance between retrospective tissue analyses and prospective phase 1 clinical screening. Together, these results address longstanding variability in reported HLA-G prevalence and provide a benchmark for the development of companion diagnostic strategies.

A central advance of this work is the rigorous analytical validation of a clinically deployable, investigational HLA-G IHC assay. Detection of HLA-G has historically been challenging due to high sequence homology with classical HLA class I molecules, low expression levels, and spatial heterogeneity, all of which have contributed to inconsistent reporting. By demonstrating high specificity, reproducibility, and compatibility with FFPE tumor tissue, this assay overcomes key technical limitations associated with prior approaches, including those relying on tissue microarrays or less specific reagents. The high concordance between retrospective and prospective datasets further supports the robustness of this platform and its suitability for clinical implementation. These findings are consistent with previous reports emphasizing the importance of assay standardization and antibody specificity in accurately defining HLA-G expression (7, 9). Our IHC scoring strategy specifically accounted for both cytomembranous and cytoplasmic localization. Although standard IHC analysis provides valuable insights into overall tissue localization, it possesses inherent limitations in distinguishing between membrane and cytosolic distribution. Further characterization utilizing high-resolution confocal imaging or biochemical approaches, such as subcellular fractionation and Western blotting, will be essential to fully elucidate the functional availability of these HLA-G pools.

Consistent with previously published data in melanoma, we observed a distinct discordance between HLA-G mRNA and protein expression. Despite relatively high transcript levels, melanoma samples exhibited low protein prevalence by IHC. This finding aligns with established evidence suggesting that post-transcriptional, translational, or protein stability mechanisms limit surface expression in this setting (20). These findings underscore the limitations of transcriptomic data alone for target prevalence assessments and highlight the importance of protein-based data, particularly relevant for low-abundance and tightly regulated targets such as HLA-G. This discordance is particularly notable in melanoma, where relative enrichment of MHC-related, IFN-γ-responsive, and effector T-cell-associated pathways at the transcriptomic level does not translate into comparably elevated HLA-G protein expression. This finding reinforces the limitations of inferring functional protein expression from mRNA-based analyses alone and highlights the need for a more nuanced, tumor-type-specific interpretation of post-transcriptional regulation.

Beyond defining prevalence, our data provide insight into the biological context of HLA-G expression within the tumor microenvironment. HLA-G expression was consistently associated with an inflamed immune phenotype characterized by enrichment of IFN-γ signaling, antigen presentation pathways, and effector T cell signatures. Across tumor types, HLA-G expression correlated with *CD8A*, *IFNG*, and classical HLA class I genes, supporting a model in which HLA-G is induced in response to immune activation. Stratification by immune phenotype further demonstrated higher expression in inflamed or CD8-high tumors compared with immune desert phenotypes, indicating preferential expression in tumors under active immune pressure.

These findings are consistent with an adaptive immune resistance model in which IFN-γ produced by activated T cells induces expression of inhibitory molecules that attenuate anti-tumor immunity. This paradigm is well established for PD-L1 and related immune checkpoints, and emerging evidence suggests that HLA-G may be regulated through similar pathways (12). In this context, HLA-G may function as a feedback inhibitory checkpoint that limits sustained cytotoxic T cell activity. The observed co-expression of HLA-G with antigen presentation machinery further supports coordinated regulation of immune activation and immune suppression, suggesting that tumors capable of engaging T cells may simultaneously upregulate HLA-G to evade immune-mediated destruction (21).

This proposed adaptive resistance model should nonetheless be interpreted with caution, as the relationship between HLA-G expression and immune context does not follow a uniform pattern across tumor types. For example, HLA-G expression was consistently low in melanoma, an immune-inflamed tumor type generally associated with favorable responses to CPI-based therapies, whereas HLA-G expression was positively associated with an inflamed phenotype in head and neck cancer, a tumor type that is more commonly immune-excluded and associated with comparatively lower CPI response rates. These discrepancies indicate that HLA-G is unlikely to function uniformly as a single anti-tumor or pro-tumor determinant across cancers, and that its expression should be interpreted within the specific immunobiological context of each tumor type rather than as a generalizable marker of immune activation or suppression across indications. Our finding that HLA-G is highly expressed in malignancies traditionally refractory to standard ICI-based therapies, such as pancreatic and ovarian cancers, highlights its potential role as a distinct mechanism of immune evasion. Why HLA-G is preferentially upregulated in these difficult-to-treat tumors remains an important open question. Because the current study was not designed to interrogate the genomic drivers of HLA-G expression, and matched mutational data were unavailable for these cohorts, we could not determine the cell-intrinsic genetic factors underpinning this phenotype. Investigating these genomic and epigenetic drivers in CPI-resistant cohorts represents a critical direction for future research.

When evaluating target expression patterns, we observed no significant changes in HLA-G expression based on prior CPI exposure (**Supplementary Figure 2**). Biologically, this lack of modulation may reflect several underlying resistance mechanisms, including insufficient endogenous IFN-γ production or downstream defects in IFN-γ signaling components within the tumor microenvironment. However, important methodological considerations must also be factored into this interpretation. Because our analysis evaluated distinct, unpaired cohorts of pre- and post-CPI patients rather than longitudinal, paired samples from individual patients, patient-specific shifts in HLA-G expression may have been obscured by inter-patient variability. Furthermore, given the highly dynamic regulation of HLA-G, our reliance on samples collected relatively far downstream of CPI therapy (median post-CPI: 65 days) introduces a kinetic limitation, making it possible that the optimal window required to capture transient, treatment-induced changes was missed.

In this study, it was not feasible to evaluate the relationship between target expression and clinical efficacy due to the fact that the study was terminated early with only 3 patients treated during dose escalation prior to achieving anticipated therapeutic doses. Furthermore, similar to other T cell engagers, target expression alone may not be the sole determinant driving response, and additional tumor microenvironment features including T cell infiltration and function are likely critical. Further studies will be required to clarify the functional role of HLA-G across tumor types. The spatial heterogeneity observed in HLA-G protein expression further supports this model. The focal and patchy distribution of HLA-G within tumors suggests that its expression is driven by localized microenvironmental cues, such as cytokine gradients or immune cell infiltration, rather than constitutive oncogenic signaling. This has important implications for biomarker assessment and therapeutic targeting, emphasizing the need for whole-tissue evaluation and raising the possibility that regional immune dynamics may influence treatment response.

A key translational challenge for HLA-G-targeted therapies is the definition of clinically meaningful expression thresholds. In our phase 1 study, an inclusive threshold of at least 1 percent tumor cells with weak or greater staining intensity was used to enable broad patient enrollment while facilitating exploratory retrospective analyses of target threshold-efficacy relationships. Although early study termination limited the ability to correlate pre-treatment tumor target expression and T cell infiltration with clinical outcomes, the high screening positivity rate demonstrates the feasibility of implementing this assay for patient selection. Future studies incorporating functional assays, including T cell-mediated cytotoxicity in patient-derived tumor explant models, may help refine these thresholds and define biologically relevant cutoffs required for therapeutic activity.

This study has several limitations. The RNA sequencing and IHC analyses were performed in partially overlapping but distinct cohorts, which may introduce variability related to patient characteristics. Future studies incorporating orthogonal quantitative PCR-based validation across matched tissue and cell line panels may further strengthen cross-platform confidence in HLA-G expression measurements. In addition, the limited number of treated patients and early termination of the phase 1 study precludes definitive conclusions regarding the relationship between baseline tumor HLA-G expression, T cell infiltration and clinical response. Additional *in vitro* and *in vivo* mechanistic studies will be required to clarify the functional and predictive role of HLA-G across tumor types. Nevertheless, the consistency of findings across independent datasets and analytical platforms supports the robustness of the conclusions.

In summary, this study provides a comprehensive map of HLA-G expression across solid tumors. By addressing technical challenges in detection and demonstrating the importance of protein-level assessment, we establish HLA-G as a broadly expressed but tightly regulated immune checkpoint. Its association with IFN-γ-driven immune activation supports a role in adaptive immune resistance and highlights its potential as both a biomarker and therapeutic target. These findings provide a foundation for the development of companion diagnostics and inform patient selection strategies for HLA-G-directed immunotherapies.

## Data Availability

The raw data supporting the conclusions of this article will be made available by the authors, without undue reservation.
