# Supplementary material for: Multimodal characterization of HLA-G expression across solid tumors reveals transcript-protein discordance and implications for immunotherapy targeting
Source: Front Immunol. 2026 Sep 24;17:1876556. doi: 10.3389/fimmu.2026.1876556 (PMC13646069; doi:10.3389/fimmu.2026.1876556)
Supplement: Supplementary file 1 [file DataSheet1.pdf]

Supplementary Material

Multimodal Characterization of HLA-G Expression Across Solid Tumors Reveals Transcript-Protein Discordance and Implications for Immunotherapy Targeting

Supplementary Table S1. Cell lines used to assess HLA-G IHC assay specificity.

Specimen ID	Specimen type	Description	Expected HLA-G staining	Observed HLA-G staining	% HLA-G positivity
JEG3	FFPE	Uterine choriocarcinoma	Positive	Positive	95
U266	FFPE	Myeloma	Negative	Negative	0
HEL	FFPE	Leukemia	Negative	Negative	0
HEK293	FFPE	Kidney	Negative	Negative	0
K562	FFPE	Leukemia	Negative	Negative	0
HEK293T	FFPE	Parent	Negative	Negative	0
HEK293T-HLA-A	FFPE	HLA-A transfected	Negative	Negative	0
HEK293T-HLA-B	FFPE	HLA-B transfected	Negative	Negative	0
HEK293T-HLA-C	FFPE	HLA-C transfected	Negative	Negative	0
HEK293T-HLA-E	FFPE	HLA-E transfected	Negative	Negative	0
HEK293T-HLA-F	FFPE	HLA-F transfected	Negative	Weak	8
HEK293T-HLA-G	FFPE	HLA-G transfected	Positive	Positive	50

FFPE, formalin-fixed paraffin-embedded; HLA, human leukocyte antigen; IHC, immunohistochemistry.

Supplementary Table S2. HLA-G expression assessed by IHC in normal human tissue.

No.	Age	Sex	Organ	Diagnosis	0	1+	2+	3+	H-score	% HLA-G positive membrane
1	35	F	Skin	Normal	97	3	0	0	3	0
2	46	F	Breast	Normal	100	0	0	0	0	0
3	53	F	Spleen	Normal	70	20	6	4	44	0

4	50	F	Lymph node	Normal	100	0	0	0	0	0
5	57	M	Skeletal muscle	Normal	100	0	0	0	0	0
6	39	M	Lung	Normal	100	0	0	0	0	0
7	67	M	Heart	Normal	100	0	0	0	0	0
8	69	M	Salivary gland, sublingual	Normal	80	20	0	0	20	20
9	70	M	Liver	Normal	100	0	0	0	0	0
10	65	M	Gallbladder	Normal	100	0	0	0	0	0
11	73	F	Pancreas	Normal	100	0	0	0	0	0
12	22	M	Tonsil	Normal	90	7	3	0	13	0
13	76	F	Esophagus	Normal	100	0	0	0	0	0
14	63	M	Stomach, antrum	Normal	100	0	0	0	0	0
15	56	M	Stomach, fundus	Normal	100	0	0	0	0	0
16	40	F	Stomach, muscle layer	Normal	100	0	0	0	0	0
17	44	M	Small bowel	Normal	100	0	0	0	0	0
18	62	M	Appendix	Normal	100	0	0	0	0	0
19	73	M	Colon	Normal	100	0	0	0	0	0
20	64	M	Rectum	Normal	98	2	0	0	2	0
21	49	M	Kidney, cortex	Normal	100	0	0	0	0	0
22	54	M	Kidney, medulla	Normal	99	1	0	0	1	0
23	82	M	Urinary bladder	Normal	100	0	0	0	0	0
24	32	M	Prostate	Normal	100	0	0	0	0	0
25	70	M	Testis	Normal	98	2	0	0	2	0
26	29	F	Endometrium	Normal	100	0	0	0	0	0
27	47	F	Myometrium	Normal	100	0	0	0	0	0
28	34	F	Placenta	Normal	70	0	0	30	90	30
29	44	M	Adrenal gland	Normal	97	3	0	0	3	0

30	39	M	Thyroid	Normal	100	0	0	0	0	0
31	59	M	Cerebrum	Normal	100	0	0	0	0	0
32	1	M	Cerebellum	Normal	100	0	0	0	0	0

Columns 0, 1+, 2+ and 3+ give the percentage of evaluable cells at each staining intensity. F, female; HLA-G, human leukocyte antigen-G; IHC, immunohistochemistry; M, male.

Supplementary Table S3. HLA-G gene expression in patient tumors.

Indication	No. samples	Median log2 TPM	Range (max − min)	Min log2 TPM	Max log2 TPM
RCC	45	4.802	7.833	0.291	8.123
Melanoma	27	3.229	13.195	−6.644	6.551
NSCLC	104	2.457	11.985	−4.431	7.554
Ovarian cancer	36	2.032	10.945	−6.644	4.301
Urinary-system cancer	75	1.910	12.654	−6.644	6.010
Pancreatic cancer	17	1.732	6.685	−1.907	4.777
CRC	81	1.607	9.023	−3.482	5.540
Other GI cancer	162	1.582	14.356	−6.644	7.712
Uterine cancer	28	1.458	9.410	−3.377	6.033
H&N cancer	79	1.106	16.458	−6.644	9.815
Breast cancer	27	0.605	9.792	−6.644	3.149

The tumor-type composition of Supplementary Table S3 (RNA sequencing cohort; approximately 600 patients across eleven solid tumor indications from early-phase clinical trials) differs from that of Tables 1–3 (IHC prevalence cohort with 252 retrospective tumor samples across six indications and 40 prospectively screened phase 1 patients across five indications), reflecting the distinct patient populations and data sources underlying the transcript- and protein-level analyses, as described in the Materials and Methods.

CRC, colorectal cancer; GI, gastrointestinal; H&N, head and neck; NSCLC, non-small cell lung cancer; RCC, renal cell carcinoma; TPM, transcripts per million.

Supplementary Table S4. Gene signatures calculated for expression analysis.

Pathway	Genes
T cell effector	*CD8A, GZMA, GZMB, IFNG, EOMES, PRF1, CXCL9, CXCL10, TBX21*
Exhausted CD8 T cell	*PDCD1, HAVCR2, LAG3, ENTPD1, CD38, TOX*
IFNg	*IDO1, CXCL10, CXCL9, HLA-DRA, STAT1, IFNG*
MHC pathway	*CALR, PSME2, TAP1, TAPBP, WSB1, PSMB9, PDIA3, PSMB8, IRF1, PSME1, CD74, PSME3*
MHC Class I	*B2M, HLA-A, HLA-B, HLA-C, HLA-E*

Pathway names correspond to the row labels shown in Figure 4A. The IFNg signature is a previously published interferon gamma-related signature (19); the remaining signatures comprise curated panels of immune-related genes selected on the basis of established literature.

Supplementary Figure Legends

Supplementary Figure S1. Analytical validation of HLA-G immunohistochemistry assay specificity.

Representative immunohistochemical staining of cell line controls used to assess assay specificity.

(A) JEG3 cells, which endogenously express HLA-G, show strong positive staining in the majority of cells, consistent with high expression. **(B)** K562 cells, lacking HLA-G expression, show no detectable staining, confirming absence of non-specific signal. **(C)** Parental HEK293T cells demonstrate no staining, consistent with lack of HLA-G expression. **(D)** HEK293T cells overexpressing HLA-F show minimal weak staining, indicating limited cross-reactivity under artificial, non-physiologic conditions. **(E)** HEK293T cells overexpressing HLA-G show robust positive staining, confirming assay sensitivity and target specificity.

The percentage of HLA-G positive cells is given in parentheses for each panel. These findings demonstrate high specificity of the assay for HLA-G, with minimal cross-reactivity against other HLA class I molecules. Scale bars, 200 µm.

Supplementary Figure S2. HLA-G gene expression by checkpoint inhibitor treatment status across tumor types.

HLA-G transcript expression stratified by checkpoint inhibitor (CPI) treatment status in patient tumors according to indication. Boxplots show log2-transformed TPM values for individual samples grouped as CPI-naive or CPI-experienced, with overlaid points representing individual tumors. The central line indicates the median, and boxes represent the interquartile range. Differences between

clinical cohorts were assessed using a two-sample Wilcoxon test across the tumor indications; all comparisons were non-significant (ns, P>0.05). Across tumor types, HLA-G gene expression is broadly comparable between CPI-naive and CPI-experienced groups, indicating that HLA-G expression is not associated with prior CPI exposure. Details of prior checkpoint inhibitor therapy are given in the Materials and Methods.
